# Self-compassion mediates the influence of mindfulness on subsequent self-forgiveness in a Polish sample

**DOI:** 10.1038/s41598-025-21198-w

**Published:** 2025-09-24

**Authors:** Sebastian Binyamin Skalski-Bednarz, Loren L. Toussaint, Patrycja Uram, Dagna Kocur, Dariusz Krok

**Affiliations:** 1 Lehrstuhl für Sozial- und Gesundheitspädagogik, Philosophisch-Pädagogische Fakultät, Katholische Universität Eichstätt-Ingolstadt, Luitpoldstraße 32, 85072 Eichstätt, Germany; 2 Institute of Psychology, Humanitas University, Sosnowiec, Poland; 3 Department of Psychology, Luther College, Decorah, IA USA; 4 Institute of Psychology, Polish Academy of Sciences, Warsaw, Poland; 5 Institute of Psychology, University of Silesia in Katowice, Katowice, Poland; 6 Institute of Psychology, University of Opole, Opole, Poland

**Keywords:** Mindfulness, Self-compassion, Self-forgiveness, Emotional resilience, Longitudinal study, Psychology, Human behaviour

## Abstract

Mindfulness is widely recognized for its effectiveness in addressing life challenges, with self-compassion increasingly explored as a potential mechanism underlying its benefits. This study examines whether self-compassion mediates the relationship between mindfulness and self-forgiveness. A three-wave longitudinal study of 164 Polish participants (aged 18–65) assessed mindfulness, self-compassion, and self-forgiveness using validated measures. Structural equation modeling tested the mediation model and alternative pathways, ensuring robust model fit through measurement invariance. Mindfulness predicted self-compassion (β = 0.43, *p* ≤ 0.001), which in turn predicted self-forgiveness (β = 0.18, *p* = 0.019), with a significant indirect effect (β = 0.08, *p* ≤ 0.001). The direct effect of mindfulness on self-forgiveness was not significant (β = − 0.04, *p* = 0.182). The mediation model demonstrated strong fit (χ²_(15)_ = 23.3, *p* = 0.078; CFI = 0.977; TLI = 0.971; RMSEA = 0.045; SRMR = 0.038). This study demonstrates that self-compassion mediates the relationship between mindfulness and self-forgiveness, helping to reduce self-criticism and build emotional resilience. The findings support incorporating mindfulness and self-compassion training into interventions for self-forgiveness. Although the sample limits generalizability, the results offer a basis for future research in clinical and diverse cultural settings.

 Emotions influence perception and behavior, often necessitating regulation to maintain adaptive functioning. *Mindfulness*, described as a receptive state of present-moment awareness cultivated through intentional, nonjudgmental attention^1^, offers a transformative approach to regulating emotions. It enables individuals to observe their emotional experiences without reactive patterns, altering their relationship with emotions and promoting self-awareness and resilience^2,3^. Evidence supports mindfulness-based interventions in reducing stress, depression, and anxiety, while enhancing quality of life^4,5^. A better understanding of these outcomes requires exploring the mechanisms through which mindfulness fosters emotional regulation and amplifies its positive effects. This discussion examines mindfulness’s impact on self-forgiveness through the mediating role of self-compassion.

Chambers et al.^6^ proposed an *integrative model of mindful emotion regulation*, emphasizing the roles of awareness and acceptance in disrupting automatic appraisal processes associated with emotional dysregulation. This model expands traditional emotion regulation frameworks by integrating dimensions beyond valence and arousal^7^. Through the cultivation of present-moment awareness, mindfulness facilitates adaptive emotional engagement, empowering individuals to respond to their emotions with intention and clarity. This process not only enhances emotional flexibility but also cultivates a deeper sense of self-awareness, contributing to overall well-being, as demonstrated by empirical studies^8–10^. From a psychological perspective, this may be due to the presence of emotional elements in the sphere of attentiveness, which requires proper regulation of feelings and moods.

Mindfulness serves as a foundation for interrupting self-criticism and emotional reactivity, fostering nonjudgmental present-moment awareness that supports self-compassion. Defined as self-kindness, shared humanity, and mindful awareness of personal struggles^11^, *self-compassion* enables individuals to approach difficulties with empathy rather than self-judgment. Mindfulness’s emphasis on acceptance and reduced overidentification with negative thoughts creates the conditions for self-compassion to emerge, facilitating a balanced and understanding perspective on challenges^12^. Theoretical conceptions describe mindfulness as involving two core components: the self-regulation of attention and an attitude of openness and acceptance toward one’s experience^13^. Building on this, Shapiro et al.^14^ emphasized the interplay of intention, attention, and attitude as mechanisms that foster reperceiving, a shift in perspective that promotes adaptive emotional engagement. Furthermore, meta-analytic findings link higher self-compassion levels to fewer psychological symptoms^15,16^. By addressing suffering with warmth and emotional regulation, self-compassion complements the acceptance nurtured by mindfulness, fostering resilience and empathy^17^. By interrupting cycles of self-criticism, mindfulness lays the groundwork for the emotional strength and kindness characteristic of self-compassion^11,18^. This dynamic interplay underscores how mindfulness predicts and supports self-compassion, equipping individuals to face challenges with emotional balance and psychological well-being.

Research consistently underscores the interrelationship between mindfulness and self-compassion, highlighting their complementary contributions to mental health. Evans et al.^19^ identified self-compassion as a mediator in the mindfulness–well-being relationship, indicating that mindfulness fosters self-compassionate attitudes that enhance its mental health benefits. Similarly, Yip et al.^20^ demonstrated that self-compassion mediates the relationship between mindfulness and occupational strain, showing that mindfulness reduces burnout and secondary traumatic stress through self-coldness (negative self-compassion qualities) and enhances compassion to clients through self-warmth (positive self-compassion qualities). Additionally, Birnie et al.^21^emphasized mindfulness’s role in cultivating self-compassion by increasing emotional awareness and acceptance. These findings are further supported by Robins et al.^22^ and Raab et al.^23^, who documented enduring self-compassion improvements in diverse populations. Shapiro et al.^24,25^ and Jazaieri et al.^26^ extended these observations to healthcare professionals, individuals with social anxiety, and parents of children with disabilities. López et al.^27^ identified specific mindfulness and self-compassion facets—such as non-reactivity and reduced self-judgment—as predictive of lower depression, anxiety, and stress levels, as well as improved well-being. Collectively, these findings suggest that mindfulness enhances self-compassion, which in turn mediates its beneficial psychological effects. These connections, at least in part, stem from the individual’s universal desire to develop inner peace, but also to deal more constructively with stress, anxiety and the challenges of modern life.

Adopting a compassionate stance toward oneself can enhance *self-forgiveness*, a vital marker of psychological health that enables individuals to move beyond self-condemnation and excessive self-criticism^28,29^. Self-forgiveness, while often seen as an emotional end-state marked by positive feelings and the resolution of internal conflict^30^, is better understood as a dynamic process^31^. This process involves acknowledging one’s actions, accepting appropriate guilt or remorse, and releasing self-directed negativity to foster healing and growth. It emphasizes accountability and reconciliation, encouraging a constructive redefinition of self-perception through meaningful connections with others and a commitment to positive change^32,33^. Both self-forgiveness and self-compassion share key elements of self-acceptance, kindness, and emotional regulation, especially in addressing guilt and adversity. These shared traits have been associated with reductions in symptoms such as anxiety and depression, highlighting their role in promoting psychological well-being and adaptive emotional functioning^34,35^. Notably, self-forgiveness is positively associated with mindfulness, indicating a connection to greater self-awareness and emotional balance^36–38^.

Models of self-forgiveness suggest that self-compassion may play an important role in this process. These frameworks describe self-forgiveness as involving the release of self-directed anger and guilt, fostering the development of self-compassion and emotional balance necessary for healing^39,40^. They also indicate that self-forgiveness may function as an internal self-regulatory mechanism for maintaining well-being, whereby individuals seek to balance positive and negative cognitive–emotional states. Extending this view, Woodyatt, Wenzel et al.^41^ demonstrated that self-compassion reduces perceived stigma and self-punitiveness, thereby facilitating self-forgiveness by helping individuals relinquish self-directed negativity and enabling emotional recovery. In their theoretical conception, this process is referred to as self-esteem restoration, which, alongside value reorientation, dynamically leads to genuine self-forgiveness and a reduction in self-punishment. Similarly, Mróz and Sornat^42^ observed that self-compassion buffers the harmful effects of shame, enabling individuals to navigate self-forgiveness more effectively by reducing tendencies toward self-condemnation. Together, these studies and theories highlight how self-compassion supports emotional regulation and addresses key barriers such as shame and stigma, thereby fostering healthier self-reconciliation and improved psychological well-being. In the context of the previously discussed mechanisms, it is plausible to assume that mindfulness provides the regulatory foundation for these dynamic processes, as Neff^11^ emphasizes that self-compassion is rooted in mindful awareness, and Chambers et al.^6^ highlight that mindfulness fosters emotional balance and reduces reactivity. Thus, self-compassion is likely to underlie the temporal interplay between mindfulness and self-forgiveness—a relationship that, to date, has not been empirically tested.

## Current study

As outlined above, the literature suggests that self-compassion may mediate the relationship between mindfulness and self-forgiveness, yet this possibility remains empirically untested. To address this gap, the present study employs a three-wave longitudinal design to examine whether mindfulness positively influences self-forgiveness through changes in self-compassion. Reports indicate that approximately 30% to 50% of individuals experience self-condemnation or harsh self-criticism over the course of their lives, often as a result of feelings of personal failure or self-blame for adverse events^43,44^. In this context, this study focuses on general tendencies and dispositional patterns of mindfulness, self-compassion, and self-forgiveness, rather than episodic experiences, to better understand mechanisms that could help individuals manage enduring emotional challenges. To provide a more rigorous test of the proposed directionality of variable relations, we also examine alternative mediation and reverse mediation models and compare their model fit to the proposed conceptual model, which is a common practice in psychological research^45–48^.

## Materials and methods

### Participants

We conducted a study in Poland, recruiting a general population sample of 231 participants for data collection across three waves in the spring of 2024: February (Wave I), April (Wave II), and June (Wave III). Participants were recruited using social media platforms, such as Facebook and X. The recruitment process was open and inclusive, with the only eligibility criteria being Polish nationality and a minimum age of 18 years. This approach ensured a broad representation of individuals from diverse demographic backgrounds within these parameters (see Table 1). The participants’ average age was 41.23 years *(SD* = 13.21), ranging from 18 to 65 years. The sample was predominantly female (61%), with most participants having completed secondary education, being employed, married or in marital relationships, and identifying with the Christian religion.

**Table 1:** Participants’ socioeconomic background (*N* = 164).

Characteristic	Percentage (%)
Gender	
Female	61
Male	39
Education	
Secondary education	48
Postsecondary education	29
Higher education	23
Employment Status	
Employed full-time	63
Employed part-time	12
Unemployed	9
Retired	8
Students	8
Marital Status	
Married or in a relationship	68
Single	25
Divorced or widowed	7
Religious Affiliation	
Christian	90
Other or no affiliation	10
Race	
White	100

To maintain participant engagement across the three waves, a structured communication strategy was implemented. At the outset, participants were informed about the longitudinal nature of the study and the importance of their continued involvement. Following each wave, participants were thanked for their participation and reminded of the schedule for subsequent data collection phases. Personalized email follow-ups and reminders were sent approximately two weeks before the start of each wave to encourage re-engagement. These messages provided a summary of the study’s purpose, clear instructions for completing the surveys, and reassurances regarding the confidentiality and secure management of their data.

All collected data, including email addresses used for communication, were securely stored on encrypted servers in compliance with European data protection regulations (GDPR). Although participant responses were anonymized through unique codes to enable linking across waves, email addresses were temporarily stored to facilitate communication. These were used exclusively for study-related purposes, such as sending reminders or updates. Participants were informed of their rights under GDPR, including the ability to access, correct, or delete their data at any point during the study.

The retention rate across the three waves was 71%, resulting in a final analyzable sample of 164 participants who completed all phases of the study. Participants who discontinued participation between waves were contacted to understand their reasons for withdrawal; however, no attempts were made to pressure them into rejoining. Retention strategies focused on emphasizing the scientific value of participants’ contributions and maintaining clear, consistent, and respectful communication throughout the study. Participants were assured that discontinuing participation would not lead to any negative consequences.

This study was approved by the university research ethics committee. Prior to participation, all individuals were provided with detailed study information, the principal investigator’s contact details, and the ethics committee’s reference number. All methods were carried out in accordance with relevant guidelines and regulations. Informed consent was obtained from all participants, ensuring their voluntary and fully informed engagement in the research process. Participants did not receive any compensation for their participation.

### Power considerations

To ensure adequate statistical power for detecting the hypothesized indirect effect in our longitudinal mediation model, we conducted an a priori power analysis using Monte Carlo simulations, following recommendations by Preacher and Hayes^49^ and Schoemann et al.^50^. Based on prior research on self-compassion as a mediator in mindfulness-outcome relationships (e.g., ^19,20^), we conservatively estimated the indirect effect size at β = 0.1. With α = 0.05, power = 0.8, and three measurement points (T1 → T2 → T3), results indicated that a minimum of 120 participants was required. Our final sample exceeded this threshold, ensuring sufficient power to detect the hypothesized mediation effect and evaluate model fit.

### Procedure

In each of the three waves, participants completed questionnaires designed to assess mindfulness, self-compassion, and self-forgiveness. Each assessment session was intentionally brief, lasting approximately 8 min, to maximize participant retention and ensure the accuracy and relevance of the collected data to the hypothesized mediation model. At the start of the study, participants provided demographic information. To maintain data quality and ensure participant attentiveness, several control measures were implemented. Response times were monitored to identify and flag responses that were unusually fast or slow. Additionally, an attention-check question (e.g., “What is 2 + 3?”) was embedded within the questionnaire to confirm that participants were engaged with the task. All collected data were carefully reviewed, and no irregularities in participant responses or attentiveness were detected, ensuring the integrity of the dataset.

### Measures

#### Trait mindfulness

Trait mindfulness was assessed using the Polish adaptation of the Freiburg Mindfulness Inventory (FMI)^51^ adapted into Polish by Radoń^52^. The FMI is a 14-item unidimensional measure that evaluates the respondent’s attention, awareness, and attitude towards present-moment experiences. Participants rated each item on a 4-point response scale ranging from 1 (*Rarely*) to 4 (*Almost always*). One item was reverse-scored, and total scores were calculated by summing responses, resulting in a possible score range of 14 to 56. Internal consistency for this measure, along with the other scales used in the study, is reported in Table 2. Sample items included: “I am open to the experience of the present moment” and “I sense my body, whether eating, cooking, cleaning, or talking.” The internal consistency coefficient of the Polish version of the FMI in the validation study^52^ was α = 0.75, whereas the values obtained in the present study are presented in Table 2.

Some studies suggest that the Freiburg Mindfulness Inventory (FMI) may be less suited for individuals without meditation experience, particularly due to potential differences in item interpretation and response patterns^53,54^. Similar psychometric concerns apply to other mindfulness measures as well^55–57^. Despite these limitations, the FMI remains one of the most widely used mindfulness assessments, producing meaningful results even among non-meditators^58,59^.

#### Self-compassion tendency

Self-compassion was measured using the 26-item Self-Compassion Scale^11^, adapted into Polish by Kocur et al.^66^. Self-compassion is defined as a general tendency to treat oneself with kindness and understanding during difficult times, acknowledging one’s shared humanity while maintaining a balanced awareness of one’s thoughts and emotions^11^. This measure evaluates six components of self-compassion: self-kindness, self-judgment, common humanity, isolation, mindfulness (in contrast to general dispositional mindfulness, it reflects balanced awareness of one’s suffering), and over-identification. Participants responded to items on a 5-point response scale ranging from 1 (*Almost never*) to 5 (*Almost always*). Total scores were calculated by summing all item responses, yielding a possible range of 26 to 130. To minimize the potential risk of multicollinearity and ensure model clarity, we used a total self-compassion score, which accounts for 95% of the meaningful variance and is empirically supported as a single, robust construct alongside its dimensions^61–63^. Sample items included: “I try to be understanding and patient toward those aspects of my personality I don’t like” and “I’m disapproving and judgmental about my own flaws and inadequacies.” In the validation study^11^, internal consistency reached α = 0.66; the coefficients from the current study are presented in Table 2.

#### Self-forgiveness

Dispositional self-forgiveness was measured using the self-forgiveness subscale from the Toussaint Forgiveness Scale^64^, adapted into Polish by Charzyńska and Heszen^65^. This subscale consists of two items, rated on a 5-point response scale (1 = *Strongly disagree* to 5 = *Strongly agree*), with higher scores indicating a greater tendency toward self-forgiveness. Both items are reverse-coded, meaning that higher raw scores reflect greater self-forgiveness. Total scores were calculated by summing responses, yielding a possible range of 2 to 10. The two items are: “I often feel that no matter what I do now, I will never make up for the mistakes I have made in the past.” and “I find it hard to forgive myself for some of the things I have done wrong.” The internal consistency coefficient for the self-forgiveness subscale of the Polish version of the measure, reported in the validation study^65^, was α = 0.67, whereas the values obtained in the present study are presented in Table 2.

### Data analyses

The statistical analysis began with an assessment of normality for all variables using the Shapiro–Wilk test and visual inspection of histograms. Descriptive statistics, including means, standard deviations, and Pearson correlations, were computed for all variables at all three time points. To examine the hypothesized longitudinal mediation, we specified a model in which mindfulness at Time 1 predicted self-compassion at Time 2, and self-compassion at Time 2 predicted self-forgiveness at Time 3. This approach enabled the evaluation of indirect effects over time, with self-compassion serving as the mediator between mindfulness and self-forgiveness.

The statistical significance of the indirect effects was assessed using the built-in estimation procedures in AMOS. Indirect effects were computed to evaluate the mediating role of self-compassion in the relationship between mindfulness and self-forgiveness. Model fit was evaluated using chi-square statistics (χ²), comparative fit index (CFI), Tucker–Lewis index (TLI), root-mean-square error of approximation (RMSEA), and standardized root mean square residual (SRMR). A non-significant chi-square statistic (*p* > 0.05) was preferred as it indicates a good model fit, although it is sensitive to sample size. Both the CFI and TLI were interpreted on the same scale, with values above 0.90 indicating acceptable fit. Acceptable thresholds for RMSEA and SRMR were set at values below 0.08^66^.

To ensure the consistency of measurement across the three waves, measurement invariance was tested. This analysis verified whether the constructs of mindfulness, self-compassion, and self-forgiveness were measured equivalently over time, ensuring that observed changes were due to actual variations in participants’ experiences rather than inconsistencies in the measurement tools^67^. Configural invariance was first assessed, testing whether the same factor structure was maintained across time points. Metric invariance (weak invariance) was then evaluated by constraining factor loadings to equality across time. Finally, scalar invariance (strong invariance) was tested by further constraining intercepts across time. A decrease in fit indices was evaluated using ΔCFI and ΔTLI, where a change of less than 0.01 indicated that invariance assumptions were not violated^68^.

All analyses were conducted using IBM SPSS Statistics (Version 29) and SPSS Amos (Version 29). Data supporting the findings are available from the corresponding author upon reasonable request, ensuring transparency and reproducibility of results.

## Results

Correlations among mindfulness, self-compassion, and self-forgiveness across all three time points are presented in Table 2. Mindfulness demonstrated large positive correlations across time points, ranging from *r* = 0.73 (*p* ≤ 0.001) between T1 and T3 to *r* = 0.79 (*p* ≤ 0.001) between T1 and T2. Similarly, self-compassion exhibited large correlations across time points, with values ranging from *r* = 0.61 (*p* ≤ 0.001) between T1 and T3 to *r* = 0.72 (*p* ≤ 0.001) between T1 and T2. Self-forgiveness also demonstrated strong stability over time, with correlations of *r* = 0.65 (*p* ≤ 0.001) between T1 and T3 and *r* = 0.75 (*p* ≤ 0.001) between T2 and T3.

**Table 2:** Descriptive statistics and correlations (*N* = 164).

Variable		M (SD)	α	Skewness	Kurtosis	1.	2.	3.	4.	5.	6.	7.	8.	9.
1. Mindfulness T1		35.85 (6.3)	0.89	0.3	0.44	—								
2. Mindfulness T2		35.55 (5.71)	0.91	–0.09	–0.23	0.79^***^								
3. Mindfulness T3		36.15 (6.12)	0.9	0.12	–0.45	0.73^***^	0.77^***^	—						
4. Self-Compassion T1		72.67 (16.86)	0.95	0.11	0.14	0.56^***^	0.57^***^	0.59^***^	—					
5. Self-Compassion T2		73.84 (14.35)	0.94	–0.48	0.44	0.57^***^	0.58^***^	0.54^***^	0.72^***^	—				
6. Self-Compassion T3		78.99 (13.21)	0.94	–0.73	0.89	0.39^***^	0.52^***^	0.52^***^	0.61^***^	0.71^***^	—			
7. Self-Forgiveness T1		5.55(2.32)	0.75	0.17	–0.80	0.31^***^	0.27^***^	0.31^***^	0.45^***^	0.39^***^	0.38^***^	—		
8. Self-Forgiveness T2		5.61 (2.17)	0.76	–0.08	–0.98	0.23^**^	0.27^***^	0.31^***^	0.42^***^	0.54^***^	0.46^***^	0.69^***^	—	
9. Self-Forgiveness T3		5.91 (2.35)	0.74	–0.15	–0.99	0.23^**^	0.23^**^	0.3^***^	0.42^***^	0.48^***^	0.27^***^	0.65^***^	0.75^***^	—
Age						0.17^*^	0.16^*^	0.18^*^	0.19^*^	0.2^**^	0.17^*^	0.18^*^	0.17^*^	0.17^*^
Sex (0 = female, 1 = male)						0.11	0.9	0.12	0.07	0.05	0.09	–0.05	0.01	–0.06

^*^p ≤ 0.05, ^**^p ≤ 0.01, ^***^p ≤ 0.001.

Cross-construct correlations were moderate to large. At T1, mindfulness correlated strongly with self-compassion (*r* = 0.56, *p* ≤ 0.001) and moderately with self-forgiveness (*r* = 0.31, *p* ≤ 0.001). At T1, self-compassion and self-forgiveness also demonstrated a moderate positive correlation (*r* = 0.45, *p* ≤ 0.001). Similar patterns were observed across subsequent time points. Across all time points, mindfulness consistently showed stronger correlations with self-compassion than with self-forgiveness.

### Measurement invariance

Measurement invariance was assessed to ensure that mindfulness, self-compassion, and self-forgiveness were measured equivalently across three time points. As shown in Table 3, the configural model demonstrated good fit, indicating that the factor structure was stable over time. When factor loadings were constrained to equality across time (metric invariance), the model fit remained satisfactory (ΔCFI = − 0.003), supporting the equivalence of factor loadings and confirming that the constructs retained consistent meaning across time points. Adding constraints for scalar invariance, which assumes equal intercepts across time, resulted in a minor decrease in model fit (ΔCFI = − 0.008). Nonetheless, the fit indices remained within acceptable thresholds, supporting the comparability of latent means across time. These findings establish configural, metric, and scalar invariance, confirming consistent measurement of the constructs over time and providing a robust foundation for subsequent analyses, including the examination of longitudinal mediation effects.

**Table 3:** Fit indices for measurement invariance testing (*N* = 164).

Model	χ²	df	CFI	TLI	RMSEA (90% CI)	SRMR	ΔCFI
Configural	94.56	48	0.975	0.963	0.045 (0.034, 0.056)	0.038	—
Metric	103.21	54	0.972	0.961	0.047 (0.036, 0.058)	0.041	–0.003
Scalar	118.75	60	0.964	0.954	0.050 (0.039, 0.062)	0.044	–0.008

### Mediation analysis

**Fig. 1:**
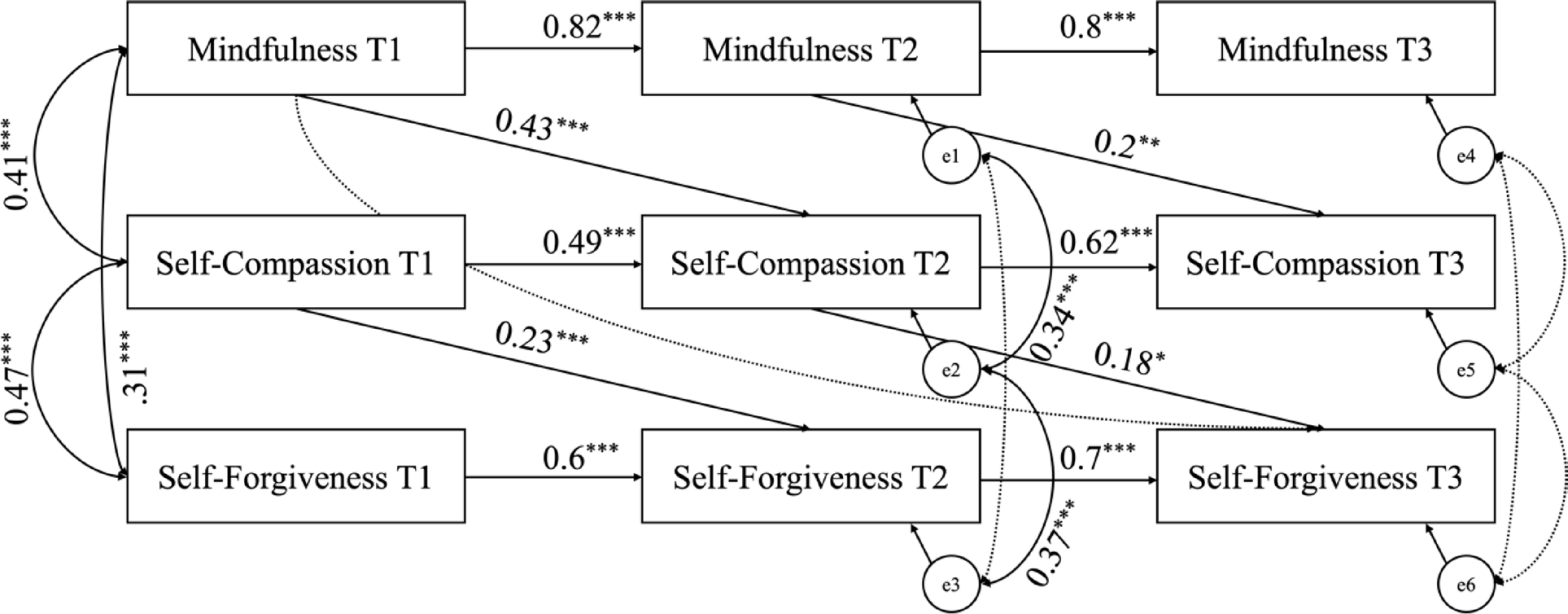
Mediating Role of Self-Compassion in the Relationship Between Mindfulness and Self-Forgiveness Across Three Time Points Note: Standardized regression coefficients (β) are reported for significant paths (^*^p ≤ 0.05, ^**^p ≤ 0.01, ^***^p ≤ 0.001). The model controls for the effect of age on self-forgiveness at T3 (β = 0.04,*p = 0.006). Sex was not included as a covariate because it showed no significant associations in the bivariate correlation matrix (see* Table 2*).*

In the mediation analysis, SEM with the maximum likelihood estimation method was employed to test the proposed hypotheses. A three-wave cross-lagged panel analysis was conducted to explore the reciprocal relationships among mindfulness, self-compassion, and self-forgiveness at three distinct time points (see Fig. 1). This approach allowed for the assessment of directional relationships over time while controlling for the prior levels of each variable. The analysis included significant cross-lagged effects and autoregressive paths for each variable at T1, T2, and T3, as well as covariances among variables at all three time points.

The results indicated that mindfulness at T1 significantly predicted self-compassion at T2 (β = 0.43, *p* ≤ 0.001), while self-compassion at T2 significantly predicted self-forgiveness at T3 (β = 0.18, *p* = 0.019). The total effect of mindfulness at T1 on self-forgiveness at T3 was also significant (β = 0.17, *p* < 0.001). Furthermore, mindfulness at T1 had an indirect effect on self-forgiveness at T3 through self-compassion at T2 (β = 0.08, *p* < 0.001), confirming the mediating role of self-compassion. However, the direct effect of mindfulness at T1 on self-forgiveness at T3 was not significant (β = − 0.04, *p* = 0.182). In the model, the effect of age on self-forgiveness at T3 was additionally controlled, revealing a small but significant positive association (β = 0.04, *p* = 0.006). The model’s fit indices demonstrated strong overall fit (χ²_(15)_ = 23.3, *p* = 0.078; CFI = 0.977; TLI = 0.971; RMSEA = 0.045 [90% CI: 0.034, 0.056]; SRMR = 0.038), confirming the appropriateness of the hypothesized model.

Additionally, alternative models were tested to evaluate the robustness of the hypothesized mediation pathway. In one alternative model, self-forgiveness was tested as a mediator of the relationship between mindfulness and self-compassion. However, this model demonstrated poor fit: χ²_(15)_ = 100.11, *p* < 0.001; CFI = 0.896; RMSEA = 0.085 [90% CI: 0.073, 0.098]; SRMR = 0.075. Another model hypothesized that mindfulness mediated the influence of self-compassion on self-forgiveness, which also showed inadequate fit: χ²_(15)_ = 110.19, *p* < 0.001; CFI = 0.874; RMSEA = 0.091 [90% CI: 0.079, 0.103]; SRMR = 0.083. Similarly, a third model, where mindfulness mediated the relationship between self-forgiveness and self-compassion, yielded poor fit indices: χ²_(15)_ = 117.11, *p* < 0.001; CFI = 0.86; RMSEA = 0.095 [90% CI: 0.083, 0.108]; SRMR = 0.089. Another tested model proposed that self-forgiveness mediated the relationship between mindfulness and self-compassion, but it also failed to meet acceptable thresholds: χ²_(15)_ = 112.84, *p* < 0.001; CFI = 0.868; RMSEA = 0.093 [90% CI: 0.081, 0.106]; SRMR = 0.087. Lastly, a model in which self-forgiveness mediated the relationship between self-compassion and mindfulness was examined. Although this model yielded the best fit among the alternative models, its fit indices remained insufficient: χ²_(15)_ = 93.41, *p* < 0.001; CFI = 0.899; RMSEA = 0.081 [90% CI: 0.074, 0.088]; SRMR = 0.073.

## Discussion

This study traced the dynamic process by which mindfulness contributes to self-forgiveness via self-compassion, relying on a three-wave design to capture changes over time. Situated within the growing literature on the benefits of self-forgiveness^31,39,69^, the findings underscore the interconnected roles of mindfulness and self-compassion in psychological well-being. Specifically, the results highlight their potential to promote emotional regulation and adaptive coping in the context of daily adversities, offering valuable insights into how these constructs support mental health in a non-clinical population. Importantly, the achieved sample size provided sufficient statistical power to detect the hypothesized effects, thereby supporting the robustness of the findings. By employing a longitudinal design, this research adds temporal evidence to the mindfulness–self-compassion–self-forgiveness pathway, moving beyond the cross-sectional associations that have dominated previous studies.

The findings supported the hypothesized mediation model, demonstrating that mindfulness influenced self-forgiveness entirely through its effect on self-compassion. When controlling for the indirect pathway, the direct effect of mindfulness on self-forgiveness was non-significant, indicating full mediation. These results align with previous studies that emphasize mindfulness’s role in fostering self-compassion^19,21^, as mindfulness promotes present-moment awareness and acceptance, thereby interrupting cycles of self-criticism and emotional reactivity^11,12^. By cultivating self-compassion, mindfulness enables individuals to approach personal shortcomings with kindness and understanding, ultimately fostering self-forgiveness^40,41^. Self-compassion, through personal insight, may also provide mental tools for objective analysis of one’s own behavior related to past transgressions, which can consequently lead to forgiveness of potential traumas or resentments. While prior research has identified self-compassion as a mediator between mindfulness and other outcomes, such as well-being^19^ and occupational strain^20^, this study is the first to demonstrate its mediating role in the relationship between mindfulness and self-forgiveness. Furthermore, the findings align with theoretical and therapeutic models of self-forgiveness, which highlight the importance of self-compassion and emotional regulation in reducing guilt and fostering a shift from self-condemnation to self-acceptance^39,40^.

The results also align with the hedonic pathway to self-forgiveness proposed by Woodyatt et al. ^70^, wherein self-compassion facilitates emotional recovery by alleviating self-directed anger and guilt. This pathway conceptualizes self-forgiveness as a state of enhanced subjective well-being, marked by increased positive self-regard and reduced negative emotions such as shame and guilt. Self-compassion enables individuals to release self-condemnation and develop the emotional resilience needed to reconcile with past actions^12,71,72^. In addition to the hedonic pathway, Woodyatt et al. ^70^ also described a eudaimonic path, which emphasizes reaffirming violated values and addressing moral and social identity threats. This view finds empirical support in previous research demonstrating that self-compassion plays a positive role in psychological well-being through positively influencing interpersonal memories and the experience of feeling safe and secure in current social relationships^73^. In this context, the presence of an attitude of self-forgiveness is an additional factor conducive to achieving a state of well-being. While this study provides longitudinal support for the hedonic pathway, it further extends these findings by highlighting how mindfulness enhances self-awareness and acceptance, fostering self-compassion as a means to support emotional recovery and self-forgiveness. In doing so, it offers evidence that self-compassion is not merely a correlate but a necessary mechanism through which mindfulness translates into deeper self-reconciliation processes.

Alternative mediation models were also tested, including variations where mindfulness was examined as a potential mediator. However, these models exhibited weaker fit indices, indicating they were not supported compared to the hypothesized pathway. While this study focused on evaluating indirect effects within the specified mediation model, the lack of support for alternative pathways does not rule out the possibility of causal relationships between individual constructs. Theoretical frameworks and existing research suggest that mindfulness and self-compassion are closely interrelated and may influence one another. For example, mindfulness enhances nonjudgmental awareness, which serves as a foundation for self-compassion^12,27^. In turn, self-compassion may strengthen mindfulness by reducing emotional reactivity and fostering balanced attention to personal challenges^11^. Although these ideas are grounded in theory and cross-sectional findings, they underscore the complexity of these constructs. Future studies could further clarify their interplay across different contexts and populations, but such analyses extend beyond the scope of the present research.

### Practical implications

Programs designed to promote self-forgiveness, such as the “Restore: The Journey Toward Self-Forgiveness” intervention, have shown efficacy in reducing self-condemnation and improving mental well-being, as demonstrated in both non-clinical and clinical trials (e.g.^29,74^). This structured program utilizes reflective exercises and writing tasks to support emotional healing and strengthen psychological resilience. Building on these outcomes, our findings suggest that incorporating mindfulness-based practices, such as meditation or awareness exercises, could further enhance their effectiveness. Mindfulness fosters nonjudgmental awareness and acceptance, which can disrupt cycles of self-criticism and facilitate emotional recovery^75^. Additionally, integrating self-compassion training—focused on cultivating kindness toward oneself and recognizing shared human experiences—could further reinforce emotional resilience and support long-term psychological recovery^76^. Combining these evidence-based approaches within self-forgiveness interventions holds significant promise for addressing a variety of psychological and emotional challenges.

### Limitations and future directions

This study examined dispositional traits within a healthy, non-clinical population, which limits its direct applicability to clinical settings. While the findings suggest that mindfulness and self-compassion foster self-forgiveness and relatively enhance well-being amidst daily adversities, their relevance to addressing severe psychological challenges, such as profound self-condemnation or guilt, remains unclear. Furthermore, the exclusive use of a Polish sample constrains the generalizability of the results to other cultural contexts, highlighting the need for replication across more diverse populations. Another limitation lies in the reliance on self-reported measures, which reflect declared tendencies rather than actual behaviors. This methodology may not adequately capture how individuals respond to intense self-condemnation in real-life scenarios. Prior research has indicated that dispositional forgiveness does not always align with situational forgiveness, particularly in the context of forgiving others^77,78^. Similar discrepancies may exist for self-forgiveness, warranting further investigation through experimental or observational designs. Future studies should explore how mindfulness and self-compassion dynamically interact in clinical populations or under conditions of heightened self-critical distress. Longitudinal and intervention-based designs could also clarify how these constructs influence one another and contribute to state-specific self-forgiveness processes over time. Additionally, this study extends the understanding of self-forgiveness by providing a foundation for developing structured programs aimed at promoting emotional healing through the integration of mindfulness, self-compassion, and self-forgiveness. Testing the efficacy of such programs through randomized trials would offer robust evidence of their impact and further inform the field of forgiveness-focused interventions.

## Conclusions

This study highlights the mediating role of self-compassion in the relationship between mindfulness and self-forgiveness. Findings from this longitudinal research suggest that reducing self-criticism and enhancing emotional resilience allow mindfulness and self-compassion to work together in promoting psychological well-being and adaptive coping strategies. These results emphasize the potential value of integrating elements of mindfulness and self-compassion into interventions aimed at addressing self-condemnation and encouraging self-forgiveness. While the study’s sample limits its applicability to diverse populations, its insights lay a solid groundwork for future research in clinical and culturally diverse settings.

## Data Availability

The data supporting the findings of this study are available upon reasonable request from the corresponding author, Sebastian Binyamin Skalski-Bednarz.
